# Prediction of Active Microwave Backscatter Over Snow-Covered Terrain Across Western Colorado Using a Land Surface Model and Support Vector Machine Regression

**DOI:** 10.1109/jstars.2021.3053945

**Published:** 2021-01-22

**Authors:** Jongmin Park, Barton A. Forman, Hans Lievens

**Affiliations:** Universities Space Research Association, Columbia, MD 21046 USA, and also with the NASA Goddard Space Flight Center, Greenbelt, MD 20771 USA; Department of Civil and Environmental Engineering, University of Maryland, College Park, MD 20742 USA; Department of Earth and Environmental Sciences, KU Leuven, 3000 Leuven, Belgium, and also with the Department of Environment, Ghent University, 9000 Ghent, Belgium

**Keywords:** Land surface model, NASA land information system (LIS), snow-covered terrain, support vector machine (SVM), synthetic aperture radar (SAR)

## Abstract

The main objective of this article is to develop a physically constrained support vector machine (SVM) to predict C-band backscatter over snow-covered terrain as a function of geophysical inputs that reasonably represent the relevant characteristics of the snowpack. Sentinel-1 observations, in conjunction with geophysical variables from the Noah-MP land surface model, were used as training targets and input datasets, respectively. Robustness of the SVM prediction was analyzed in terms of training targets, training windows, and physical constraints related to snow liquid water content. The results showed that a combination of ascending and descending overpasses yielded the highest coverage of prediction (15.2%) while root mean square error (RMSE) ranged from 2.06 to 2.54 dB and unbiased RMSE ranged from 1.54 to 2.08 dB, but that the combined overpasses were degraded compared with ascending-only and descending-only training target sets due to the mixture of distinctive microwave signals during different times of the day (i.e., 6 A.M. versus 6 P.M. local time). Elongation of the training window length also increased the spatial coverage of prediction (given the sparsity of the training sets), but resulted in introducing more random errors. Finally, delineation of dry versus wet snow pixels for SVM training resulted in improving the accuracy of predicted backscatter relative to training on a mixture of dry and wet snow conditions. The overall results suggest that the prediction accuracy of the SVM was strongly linked with the first-order physics of the electromagnetic response of different snow conditions.

## I. Introduction

Snow serves as a water tower that stores winter precipitation and discharges it through snowmelt [1]. It supplies freshwater to more than 1.2 billion people (approximately one-sixth of the world’s population) for agricultural and human usage [2]–[4]. Snowmelt during the springtime significantly increases streamflow, which influences the terrestrial hydrological cycle [5]. Snow also exerts a critical control on the terrestrial energy cycle. That is, the relatively high albedo of snow results in reflecting much of the incoming solar radiation that dictates much of the Earth’s energy balance [6], [7]. Snowmelt also directly influences soil moisture that further controls the partitioning of net radiation at the Earth’s surface [8]. Suffice it to say that snow is an important resource across the globe and that it merits scientific study so that this resource may be better characterized, managed, and preserved.

Quantification of snow water equivalent (SWE) and snow depth has commonly been conducted using ground-based networks [e.g., Snow Telemetry (SNOTEL), Global Surface Summary of the Day (GSOD), and National Weather Service (NWS) Cooperative Stations (COOP)]. These observations have been widely used for evaluating snowpack properties (e.g., SWE and snow depth) from remote sensing retrievals as well as from land surface models (LSMs) [9], [10]. However, *in situ* observations have limitations in capturing the spatio-temporal dynamics of snow mass given the sparsity of those observational networks. Snow mass estimates from a LSM is one alternative to overcoming the spatio-temporal limitations of ground-based observations. Many LSMs including the community land model [11], variable infiltration capacity (VIC) [12], and NOAH-multi parameterization (NOAH-MP) [13] have been used for snow estimation. However, snow estimates from LSMs contain their own uncertainties related to model parameterization, model structure, boundary conditions, and initial conditions [9], [14].

Space-borne instrumentation has been applied as an alternative to conventional measurements in order to quantitatively characterize the physical properties of snow. Satellite imagery from visible and infrared sensors is primarily used for mapping snow cover area (SCA) and snow cover fraction (SCF) based on the high albedo of snow relative to other natural surfaces [15], [16]. It is known as one of the most intuitive approaches to obtain SCA at a fine spatial resolution even though it has limitations in complex terrain, dense vegetation, and difficulty in discriminating clouds from snow [17]. Moreover, difficulty in converting SCF to SWE using a snow depletion curve has severe limitations in the optical retrieval of snow mass [18].

Active microwave remote sensing is another space-based option for estimating snowpack properties (e.g., snow cover, snow depth, SWE, snow density, and snow liquid water content) by measuring the intensity of the returned signal emitted by the radar. The amount of signal return (also known as backscatter) is highly sensitive to the liquid water content inside the snowpack, which is a result of the significant difference between the ice and water dielectric constants in the microwave spectrum and offers a relatively finer spatial resolution (compared to passive microwave (PMW) radiometry) over wide areas regardless of weather and daylight conditions [19]. Previous studies showed the potential capability of obtaining snowpack information using SAR measurements at Ku-band [20]–[24], X-band [25]–[30], and C-band, including RADARSAT [31]–[33], RADARSAT2 [19], [34]–[36], the European Remote Sensing satellite (ERS)-1/2 [37]–[41], ENVISAT [40], [42]–[44], and Sentinel-1 [45]–[50].

In virtue of the increased availability of SAR imagery for retrieving snowpack information, machine learning (ML) techniques have been widely applied to retrieve snow cover properties [44], [49], [51]–[53]. For instance, Tsai *et al.* [49] utilized a random forest technique to classify dry snow versus wet snow conditions using Sentinel-1 imagery in conjunction with ancillary information (e.g., digital elevation model, land cover classification). Longepe *et al.* [53] was the first to use a support vector machine (SVM) to classify snow cover extent using the phased array-type L-band synthetic aperture radar (PALSAR). Overall results confirmed that snow cover classification based on the ML technique yielded reasonable accuracy when compared with SCA retrieved from optical imagery. In addition to the problem of classification, ML has the ability to map geophysical model states into observation space, which makes ML a suitable candidate for use as an observation operator within a data assimilation (DA) framework [54]–[58]. The main objective of this article is to assess the potential of a ML algorithm, specifically SVM regression in time, to accurately predict C-band backscatter over snow-covered land. The accuracy of the predicted backscatter is diagnosed in terms of the selection of training targets, training windows, and physically based constraints. Such an analysis is a necessary precursor to a proposed DA framework to be completed in a follow-on study.

## II. Study Area, Data, and Model

### A. Study Area

**Fig. 1.**
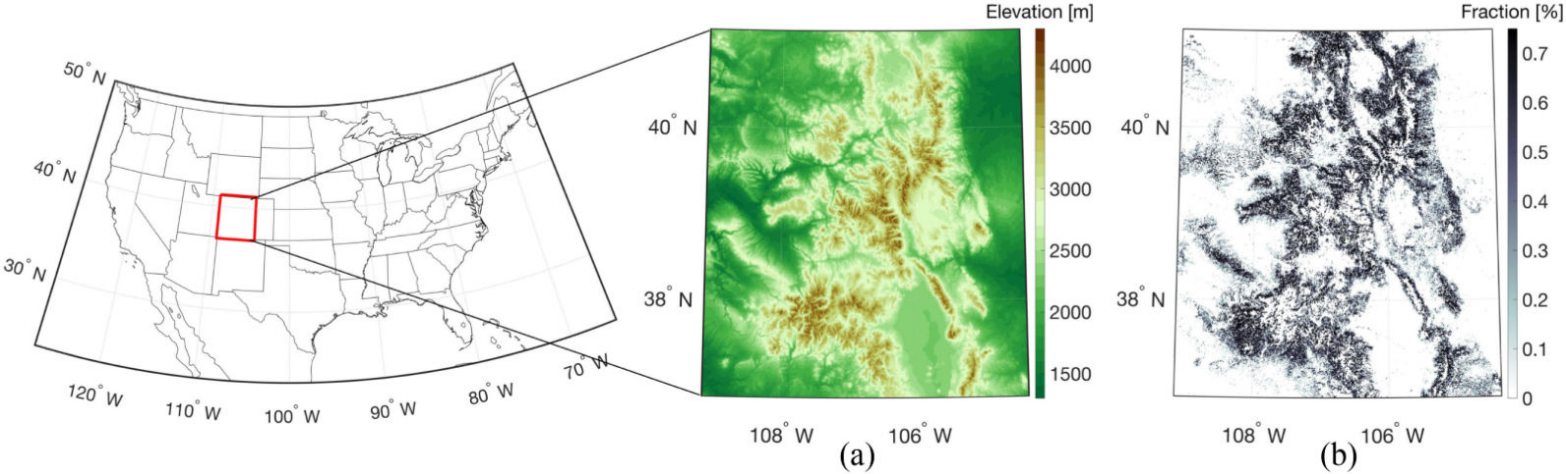
Maps of the study domain showing (a) elevation obtained from 30-m resolution SRTM data and (b) forest cover fraction obtained from the global forest cover dataset [59].

The study domain selected here is Western Colorado within the latitude of 36.875° N and 41.125° N and longitude of 104.375°W and 109.125°W (Fig. 1). The study area contains three national forests in the southern Rocky Mountains (i.e., San Juan, Rio Grande, and Grand Mesa) and has an elevation that ranges from 1314 to 4125 m with over 60% of the total study area at elevations higher than 2250 m. The dominant forest cover in the study domain is lodgepole pine, classified as an evergreen conifer, according to the forest type map established by the United States Forest Service.^1^ This study domain was selected because it contains a variety of snow conditions (e.g., deep versus shallow snow; dry versus wet snow; snow with overlying vegetation; and snow without) across a range of different topographies and land-cover types. Furthermore, the domain helps leverage the NASA SnowEx campaign, which is a multiyear airborne and ground-based snow campaign with the primary objective of assessing the characteristics of snow using *in situ* and remotely sensed observations [60]. Two primary evaluation sites—Grand Mesa and Senator Beck—are located within the study area. Grand Mesa, in particular, is known as one of the largest flat-topped mountains in the world. Since SAR observations generally contain geometric distortions over complex terrain, the investigation of SAR over flat terrain can help minimize the uncertainties in the backscatter observations.

### B. Sentinel-1 Observations

Sentinel-1 is a constellation of two satellites (Sentinel-1A and −1B launched in April 2014 and April 2016, respectively) developed by the European Space Agency as a component of the European Copernicus Program [61]. Both Sentinel-1 A and Sentinel-1B carry C-band SAR sensors with a 180° orbital phase difference [62]. Sentinel-1 has a revisit frequency of 12 days for each satellite, which results in achieving a 6-day global revisit frequency between the two different satellites. However, it has an irregular data acquisition schedule over North America until 2017 due to the evolving observation (operational) scenarios, and as a result, impacts the availability of Sentinel-1 A and -IB products in these regions [46]. Among the various imaging acquisition modes and processing levels, interferometric wide (IW) swath ground-range detected (GRD) product (Table I) is utilized in this study as the primary focus of this research is to apply C-band backscatter in analyzing terrestrial snowpack characteristics.

Before applying the Sentinel-1 imagery to this analysis, it is essential to first preprocess the datasets in order to remove several sources of noise such as geometric distortion, speckle, and thermal noise [63], [64]. Accordingly, standard preprocessing steps were employed using the Google Earth Engine with an additional incidence angle normalization step following the procedures as outlined in Lievens *et al.* [46]. First, we collected all the backscatter observations per relative orbit number. Next, the backscatter observations are rescaled such that all the relative orbit numbers have the same long-term mean backscatter, on a pixel-by-pixel basis. Note that this procedure simultaneously helps to correct the biases associated with different azimuth angles (e.g., differences in viewing geometry between ascending and descending overpasses). The preprocessed Sentinel-1 imagery was then regridded (as an arithmetic average) onto a 0.01-degree equidistant cylindrical grid in order to match the resolution of the LSM used in this analysis (see Section II-C for more details).

### C. Land Information System

The NASA Land Information System (LIS) is a software framework developed at the NASA Goddard Space Flight Center to integrate a suite of LSMs, satellite observations, ground-based measurements, and data assimilation techniques in order to obtain improved posterior estimates of land surface states and fluxes [65]. Among the different LSMs, Noah-MP [13] was selected for use in this study.

Noah-MP is based on the Noah LSM and allows for multiple parameterizations for the different process formulations of land–atmospheric interactions [13]. Further, Noah-MP employs a three-layer, physically based snow model that considers melting and refreezing of snow, which results in a more accurate quantification of snow mass [13], [66]. Noah-MP was simulated over the study domain during the study period using meteorological boundary conditions provided by the Modern-Era Retrospective analysis for Research and Application, version 2 (MERRA-2) product [67]. Geophysical variables derived from Noah-MP have a spatial resolution of 0.01° and a daily temporal resolution.

## III. Methodologies

### A. Microwave Properties of Snow

Backscatter observations over snow-covered terrain are influenced by a variety of factors, including the physical properties of the snowpack (e.g., dielectric constant, snow grain size, snow liquid water content, snow density, and snow depth) as well as sensor properties (e.g., frequency, polarization, and viewing geometry) [68], [69]. Among the different snow components, snow liquid water content is regarded as one of the most important variables when considering backscatter over snow-covered terrain as dry snow and wet snow have distinctively different electromagnetic responses associated with the differences in dielectric properties between dry (ice) snow and wet (liquid water) snow [68], [70].

**Fig. 2.**
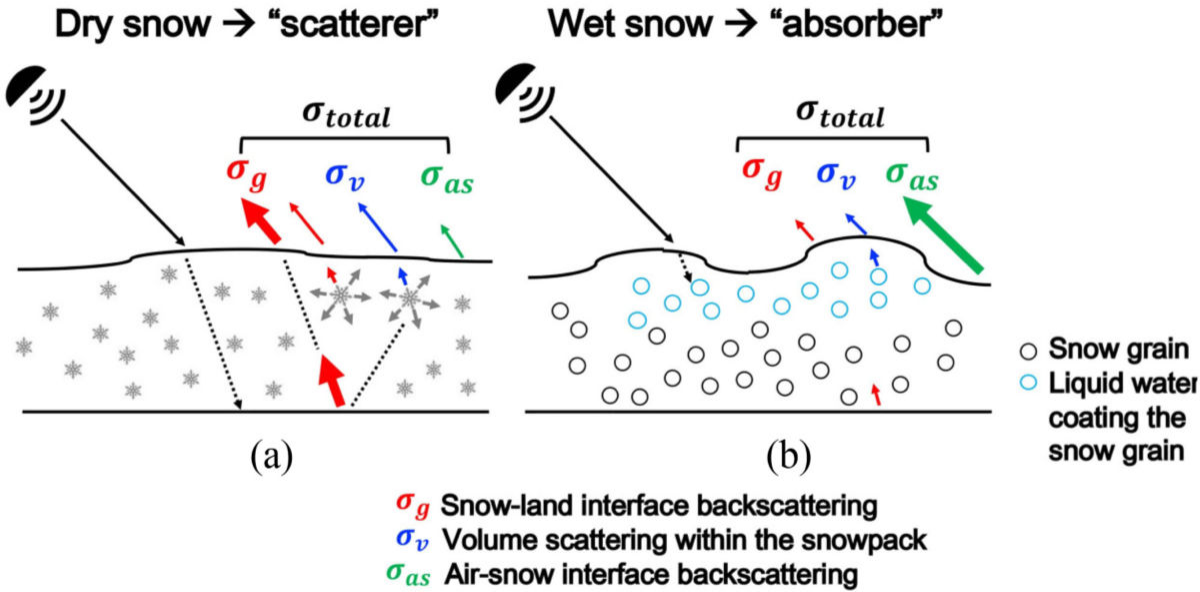
Conceptual model of active MW backscattering mechanisms during (a) dry snow conditions and (b) wet snow conditions (modified from [78]).

Fig. 2 illustrates the different scattering mechanisms over dry versus wet snow conditions. In general, dry snow acts as a scatterer of microwave (MW) radiation while wet snow behaves more as an absorber given that the presence of liquid water within the snowpack results in a large increase in permittivity [71]. During dry snow conditions, a snowpack is a mixture of air and ice. Microwave radiation can penetrate deeper into a dry snowpack (i.e., less absorption) than during wet snow conditions. Accordingly, backscatter from the underlying ground is more influential on the total observed backscatter relative to other scattering components [Fig. 2(a)]. When the snow depth or snow surface roughness increases, the influence of backscatter from within the snowpack, as well as at the air–snow interface, increases. Correspondingly, the influence of backscatter from the underlying ground is reduced [72]. In the case of wet snow, which is now a heterogeneous mixture of air, ice, and water, the MW photons cannot penetrate as deeply into the snow due to the decrease in scattering albedo and the corresponding increase in the absorption of microwave radiation associated with the existence of liquid water inside the snowpack [72], [73]. Accordingly, backscatter over wet snow is primarily dominated by backscatter at the air–snow interface in most situations [Fig. 2(b)]. However, during the ripening stage, backscatter over wet snow can also be increased due to the complex wet snow metamorphism, including an increase in snow surface roughness and an increase in the snow grain size during overnight refreezing [50], [74].

In addition, backscatter coefficients observed at different polarizations contain different amounts of information regarding terrestrial snow. In general, $\sigma_{VH}^{0}$ exhibits a gradual increase during the snow accumulation period due to the increased depolarization associated with multiple scatterings and inhomogeneities within the snowpack [21], [46], [75]. Conversely, $\sigma_{VV}^{0}$ does not exhibit significant variations, which is related to the limited sensitivity to volume scattering because backscatter from the snow-land interface often dominates [76]. During the snow ablation period, both $\sigma_{VV}^{0}$ and $\sigma_{VH}^{0}$ exhibit a relatively large sensitivity to terrestrial snow (as compared to the snow accumulation period) due to the significant absorption and reflection by liquid water content within the snowpack [45], [46].

Another important aspect affecting the backscatter coefficient is the different observation times associated with the different overpasses (i.e., ascending versus descending). Sentinel-1 has an approximate local observation time of 6 P.M. and 6 A.M. for the ascending and descending overpasses, respectively. These different observation times often reflect different snow conditions depending on the periodical cycles of melt and refreeze. For example, diurnal melting and refreezing processes result in creating internal ice layers or grain size and grain shape metamorphosis. Further, vapor pressure gradients that result from differences in snow temperature between the bottom and top of the snowpack lead to the development of depth hoar. This mixture of processes, including melting, refreezing, and sublimation, leads to a preferential increase in the backscatter for horizontal polarization as compared to vertical polarization [21], [77]. Furthermore, during the snow ablation period starting in March, roughly speaking, the top of the snow surface will have relatively wetter snow during the afternoon associated with incoming solar radiation and warmer air temperatures, which often induces surface melt. Consequently, for the ascending overpass time (~6 P.M. local time), wet snow is more likely to be found on the snow surface while any liquid water content within the snowpack has the opportunity to refreeze prior to the descending overpass (~6 A.M. local time) associated with the cold temperatures experienced during the nighttime. This difference in electromagnetic response likely contributes to the different statistical results between ascending and descending overpasses.

### B. Support Vector Machine

ML is defined as an algorithm that can “learn” a highly sophisticated, nonlinear relationship between inputs and outputs for a given physical system based on statistical inference [79]. SVM regression is a supervised ML algorithm that maps the input space into higher dimensional feature space using a kernel function [80], [81]. SVM regression has been utilized in hydrological research for spatial pattern recognition [82], [83], classification [44], [84], and temporal prediction [54], [56]–[58], [85], [86]. The study here focuses on predicting C-band backscatter over snow-covered terrain using SVM regression. The overall framework, in general, follows that of Forman and Reichle [56], although it uses different training targets and a different LSM along with different physical considerations in the context of active versus passive remote sensing of snow. A detailed description of the SVM training and prediction procedure is introduced below.

#### 1) SVM Regression:

**Fig. 3.**
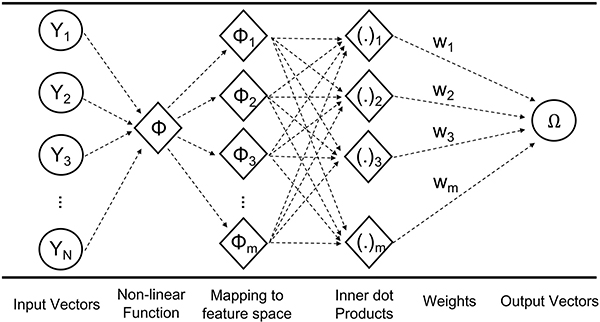
General schematic of support vector machine regression.

Fig. 3 shows the general framework of SVM regression prediction used in this study. Suppose the [1 × N] input vector (*y*) is composed of geophysical variables estimated from the LSM to characterize the physical properties of snow at a given time and location. When the input datasets are trained based on the copolarized (i.e., vertical transmit and vertical receive; $\sigma_{VV}^{0}$) and cross-polarized (i.e., vertical transmit and horizontal receive; $\sigma_{VH}^{0}$) backscatter observations from Sentinel-1, predicted backscatter at co- $\left({\hat{\sigma }}_{VV}^{0}\right)$ and cross-polarization $\left({\hat{\sigma }}_{VH}^{0}\right)$ can be computed through the nonlinear SVM expressed as follows:

(1) $$\left[\begin{array}{l} {\hat{\sigma }}_{VV}^{0}\\ {\hat{\sigma }}_{VH}^{0} \end{array}\right]=f(x)=\sum_{i=1}^{M}\left(\alpha_{i}-\alpha_{i}^{*}\right)k\left(x_{i},y\right)+\delta$$

where *M* indicates the number of available training target sets in time at a given location in space; *α*_*i*_ and $\alpha_{i}^{*}$ represent the dual Lagrangian multipliers at time *i*; and *δ* represents the bias (also known as offset) coefficients that are all defined during the training procedure. *x* is the training matrix with a size of [*M* × *N*] comprising model input vectors *y* at the times of the *M* training targets [56]. $k\left(x_{i},y\right)=\exp\left\{-\gamma {\left\|x_{i}-y\right\|}^{2}\right\}$ is a scalar radial basis kernel function (RBF) that helps map the geophysical inputs into the observation space. The rationale for choosing an RBF for the kernel in this study is based on previous research that showed an RBF yielded satisfactory performance in solving complicated, nonlinear hydrologic problems [87], [88]. The solution to (1) is calculated by employing the LIBSVM library [89], which is an open-source ML library developed by National Taiwan University. See Appendix VII for more details on the SVM regression procedure.

#### 2) SVM Inputs, Training Targets, and Outputs:

Inputs to the SVM include four geophysical variables derived from the LSM that are listed in Table II. These particular state variables were selected considering the first-order, fundamental electromagnetic response of C-band backscatter (e.g., absorption and volume scattering) over snow-covered terrain and include consideration of physically based constraints to aid the statistical learning process. The selection of these four geophysical input variables was further motivated by an exhaustive sensitivity analysis exploring a wide range of different input variables derived from Noah-MP (results not shown).

As the four variables have different ranges of magnitude, each variable was first rescaled using a scale factor in order to remove the different orders of magnitude, which will significantly influence the weights and SVM prediction capability. In addition to the LSM state variables, the Interactive Multisensor Snow and Ice Mapping (IMS) [90] binary snow cover product was used to further constrain the SVM training only when snow cover is positively detected using the visible and thermal-based snow cover estimation algorithm. Considering the selection of training targets (and outputs) for SVM, $\sigma_{VV}^{0}$ and $\sigma_{VH}^{0}$ as observed by Sentinel-1 over snow-covered terrain were trained separately based on the inherent characteristics described in Section III-A.

#### 3) Training Procedures:

An SVM was trained at each 0.01° equidistant cylindrical model grid location in order to explicitly consider the heterogeneity of regional climatology, land cover type, and topography. At each pixel, a separate SVM was generated for both ${\hat{\sigma }}_{VV}^{0}$ and ${\hat{\sigma }}_{VH}^{0}$ to predict each backscatter separately. Available Sentinel-1 observations from April 2015 to August 2016 and September 2017 to August 2018 were utilized for training, which include two complete winter seasons. Sentinel-1 observations from September 2016 to August 2017 were excluded in order to be used to evaluate the SVM prediction, which is described further in Section III-C.

There are numerous considerations when developing a physically-constrained, well-designed SVM, including parameter setups, input datasets, training targets, and training windows. Accordingly, the first experiment was conducted to analyze the influence of different training targets on SVM prediction performance. Sentinel-1 observes backscatter along ascending and descending overpasses. One of the main differences between the ascending and descending overpass is the local overpass time. Ascending measures backscatter at approximately 6 P.M. local time while descending measures backscatter at approximately 6 AM Moreover, the ascending and descending tracks have different incidence and azimuth angles in complex terrain, which leads to a different backscatter intensity [91]. Normalizing the incidence angle for both ascending and descending overpasses during the preprocessing step (previously described in Section II-B) reduces the biases associated with different azimuth angles. Hence, more available training targets (which, in general, is advantageous given a sparse training set) can be obtained by combining both the ascending and descending overpasses. However, it remains to be seen if combining different overpasses is advantageous or disadvantageous. Consequently, Sentinel-1 backscatter from the ascending node versus the descending node versus the combined overpasses was trained separately in order to explore the different impacts on SVM performance.

**Fig. 4.**
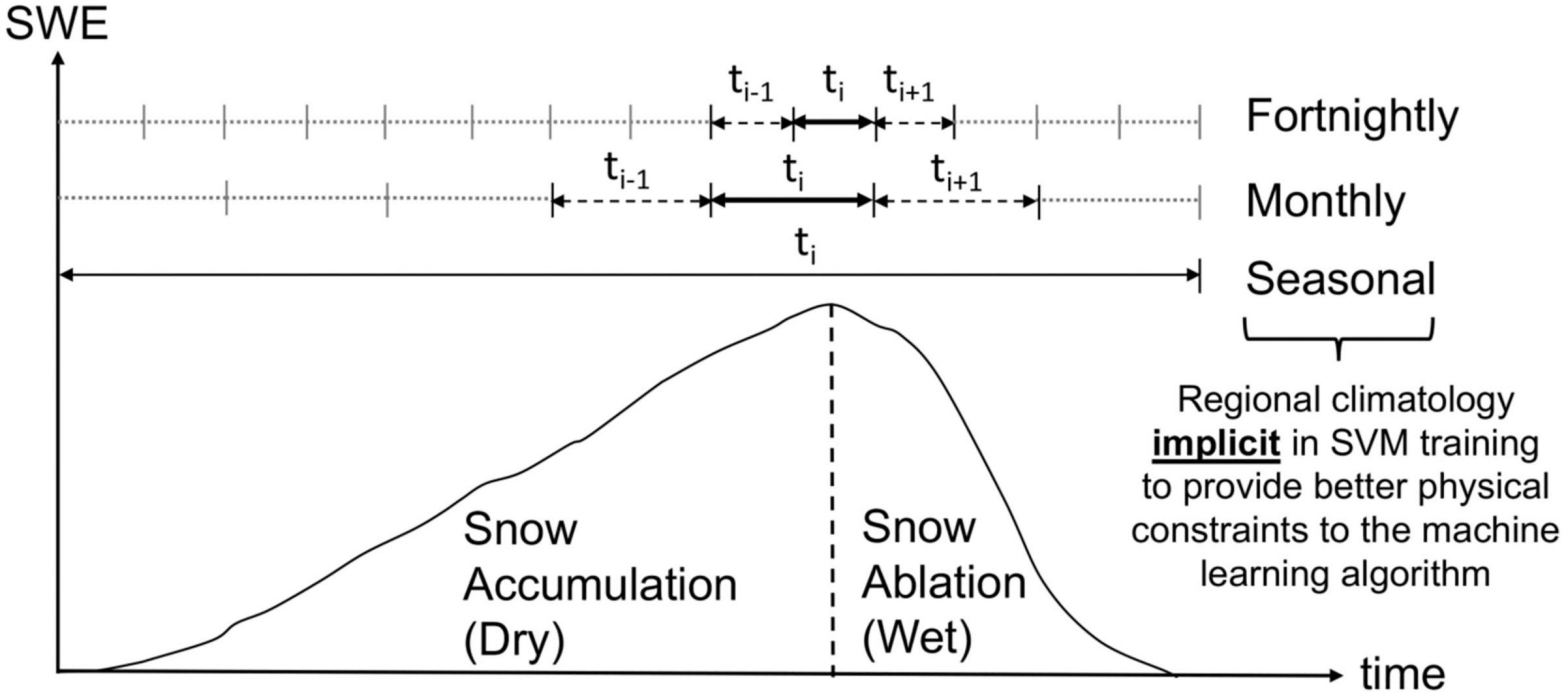
Schematic description of fortnightly versus monthly versus seasonal training approaches. Different training windows provide different degrees of wet versus dry snow delineation. Bold arrows indicate the training period and dashed arrows represent the temporal overlap. The gray dotted lines represent periods of time *not* included in the training data for the period *t*_*i*_. The shorter window provides better discrimination, but the trade-off is a less robust SVM due to fewer training targets as a function of time.

The second experiment is designed to examine the influence of different training windows on the prediction accuracy of the SVM across which to collect the Sentinel-1 training targets. Fig. 4 shows the concept of three different training windows: 1) fortnightly, 2) monthly, and 3) seasonal. The fortnightly training procedure includes 2 weeks of overlap before and after the specific fortnight (14-day period) in order to reduce temporal discontinuities between different SVMs [92]. Analogously, the monthly training period includes the month before and month after the specific month of training during the collection of the training targets. In the case of a seasonal training window, it includes the Sentinel-1 observations during the entire snow season (e.g., from September to May). The underlying rationale of the fortnightly training window is to generate a physically constrained SVM that more carefully considers the first-order control on the different electromagnetic responses from dry snow versus wet snow described in Section III-A. Thus, a shorter training window can enhance the delineation between dry versus wet snow. On the other hand, elongating the length of the training window ensures more available training data for the SVM even though there is more possibility to commingle the observations containing a different electromagnetic regime.

Finally, explicit SVM training for dry snow versus wet snow conditions was conducted separately in order to explicitly analyze the influence of snow liquid water content on SVM prediction efficacy. Considering the different first-order physics between the scattering mechanisms for dry versus wet snow conditions described in Section III-A (based on the snow liquid water content in the *a priori* LSM estimates) provides one more mechanism to explore different physically constrained training techniques to the ML procedure.

### C. Validation Approach

Predicted backscatters for both polarizations were evaluated by comparing against Sentinel-1 backscatter observations *not* used during training (i.e., from Sep 2016 to Aug 2017). This ensures that the validation dataset is independent from the Sentinel-1 datasets used for training. One of the main reasons to select Sep 2016 to Aug 2017 for validation is that this period was considered as a typical snow year for the available years in the Sentinel-1 record.

In order to quantitatively evaluate the predicted backscatter from the SVM, bias and root mean square error (RMSE) across the snow-covered grid cells were computed as

(2) $$\text{bias}\;=\frac{1}{n}\sum_{i=1}^{n}\left({\hat{\sigma }}_{\text{pol}}^{0}-\sigma_{\text{pol}}^{0}\right)$$

(3) $$\text{RMSE}=\sqrt{\frac{1}{n}\sum_{i=1}^{n}{\left({\hat{\sigma }}_{\text{pol}}^{0}-\sigma_{\text{pol}}^{0}\right)}^{2}}$$

where *n* is the number of predicted and observed backscatter values collocated at a given location in space and time and ${\hat{\sigma }}_{\text{pol}}^{0}$ (dB) and $\sigma_{\text{pol}}^{0}$ (dB) represent the predicted and observed backscatter at a given polarization, respectively. In addition, ubRMSE is utilized to identify the random error by removing the bias from RMSE as

(4) $${\mathrm{ubRMSE}}^{2}={\mathrm{RMSE}}^{2}-{\mathrm{bias}}^{2}.$$

As part of the statistical evaluation, the presence of statistically significant differences between the various domain-averaged statistics was conducted using the two-sided Wilcoxon signed rank sum test [93], which is a nonparametric hypothesis test to examine the null hypothesis that the median of two samples are not different [94]. The main reason for selecting the Wilcoxon signed rank test is that the predicted and observed backscatters are expected to be non-Gaussian, which violates the assumption for the two-sample *t*-test. For the same reason of non-Gaussianity, the correlation coefficient was not used here as an evaluation metric in favor of using other evaluation metrics instead. Furthermore, statistics during the snow accumulation (e.g., December, January, and February) and snow ablation periods (e.g., March, April, and May) were calculated separately as the delineation of dry snow versus wet snow motivates three different experiments outlined in Section III-B3.

## IV. Results

### A. Influence of Training Targets on SVM Prediction

Sentinel-1 observations from the ascending (6 p.m. local time) versus descending (6 a.m. local time) overpasses as well as the combination of the two different overpasses during Sep 2016 to Aug 2017 were utilized by examining the influence of the different training target sets on the SVM prediction efficacy. For each of the three different scenarios, four different inputs derived from LIS (see Table II) using fortnightly training were first explored. Next, predicted backscatter from the different sets of training targets was evaluated by comparing against Sentinel-1 observations *not* utilized during training. Table III summarizes the domain-averaged statistics of predicted ${\hat{\sigma }}_{VV}^{0}$ and ${\hat{\sigma }}_{VH}^{0}$ using the three different training sets for the validation period.

Results suggest that predicted backscatter using the descending-only overpasses showed the lowest absolute mean bias for both polarizations. The computed bias for the descending-only training targets ranged from −5.37 to 4.86 dB with a spatial mean of −0.74 dB for ${\hat{\sigma }}_{VV}^{0}$. Similarly, the bias ranged from −4.92 to 3.85 dB for ${\hat{\sigma }}_{VH}^{0}$ with the spatial mean of −0.82 dB. Further, the descending-only training targets exhibited the lowest mean RMSE over the study area (Table III). For the ascending-only training targets, the bias for both polarizations showed a wider range than for the descending-only training targets (i.e., from −12.3 to 11.7 dB for ${\hat{\sigma }}_{VV}^{0}$ and −5.73 to 6.96 dB for ${\hat{\sigma }}_{VH}^{0}$), which resulted in a larger magnitude of mean bias and RMSE. Among the three different training target sets, the combined dataset showed the highest RMSE and ubRMSE at both polarizations. This phenomenon is a consequence of the systematically different signals from the snowpack with respect to data acquisition time. Snow often undergoes a small amount of diurnal melting and refreezing at the snow surface depending on the diurnal temperature cycle. Thus, the descending acquisitions prior to sunrise (6 AM local time) tends to minimize wet snow conditions and are relatively dry given refreezing while ascending acquisitions following sunset (6 P.M. local time) are often relatively wet at the surface by comparison. As a result, the snowpack during the ascending overpass tends to have more wet snow in the surface layer, and in turn, results in a higher magnitude of domain-averaged bias. Moreover, a mixture of signals collected during different snow conditions and viewing geometries contained within the combined training target set resulted in a relatively larger RMSE and ubRMSE than did the other training target sets (see Supplementary Fig. S1).

**Fig. 5.**
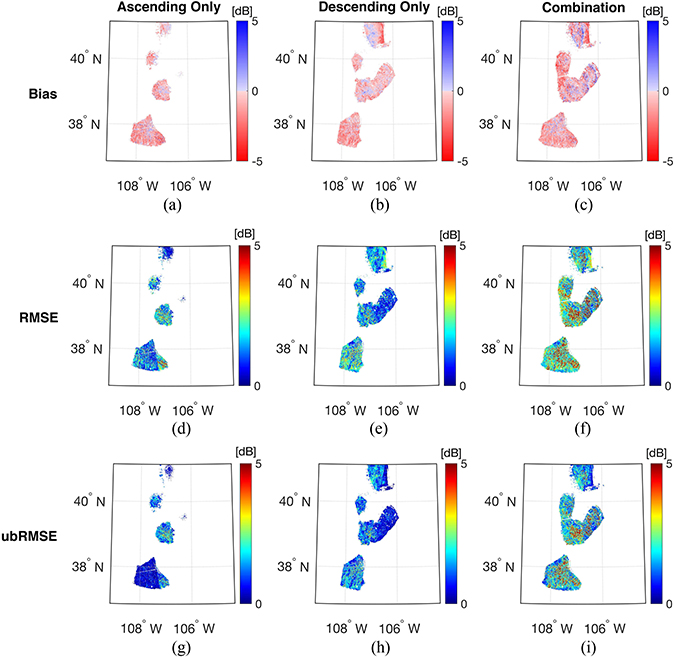
Spatial distribution of bias (top row), RMSE (middle row), and ubRMSE (bottom row) of copolarized backscatter $\left({\hat{\sigma }}_{VV}^{0}\right)$ for the validation period Sep. 2016 to Aug. 2017. The different columns represent the training target sets for ascending-only (left column), descending-only (middle column), and combination of ascending and descending (right column). The white space in each map represents the area where there are no available SVM predictions at locations due to either limited duration of snow in the LSM or insufficient Sentinel-1 observations for use during training.

Comparing Fig. 5 with the elevation map presented in Fig. 1(a), a relatively high magnitude of negative bias was observed within the elevation range of 2500–3500 m. Among the pixels that showed a bias greater than the lower decile of bias for each training target set, over 69% (77.5% for ascending-only, 71.9% for descending-only, and 69.5% for combined) of pixels were located within this elevation range. This result likely originated from the influence of vegetation on C-band backscatter in this elevation band. Comparing the spatial pattern of the elevation map with the forest cover fraction presented in Fig. 1(b), most of the pixels with high forest cover fraction are located within the range of 2500–3500 m. Huang and An-dereeg [95] mentioned that the dominant forest type for this specific elevation band is largely heterogeneous with different types of vegetation in the understory (e.g., mountain snowberry) and overstory (e.g., aspen pine). Backscatter observed from the heterogeneous forest contains little sensitivity to snow due to the vegetation-related scattering components such as multiple scatterings within the canopy and scattering from the forest floor [41], [96]. In terms of the percent spatial coverage (also known as ratio of the number of pixels with predicted backscatter within the validation period over the total number of pixels within the study domain) among the different training targets, the combination of ascending and descending overpasses yielded the highest spatial coverage followed by descending-only and ascending-only observations (Table III and Fig. 5). As the ascending and descending overpasses have different acquisition times, the concatenation of the two leads to a larger training set for a given training window. That is, the combination of the two different sets of overpasses results in obtaining more predicted backscatter at more locations across the study area.

**Fig. 6.**
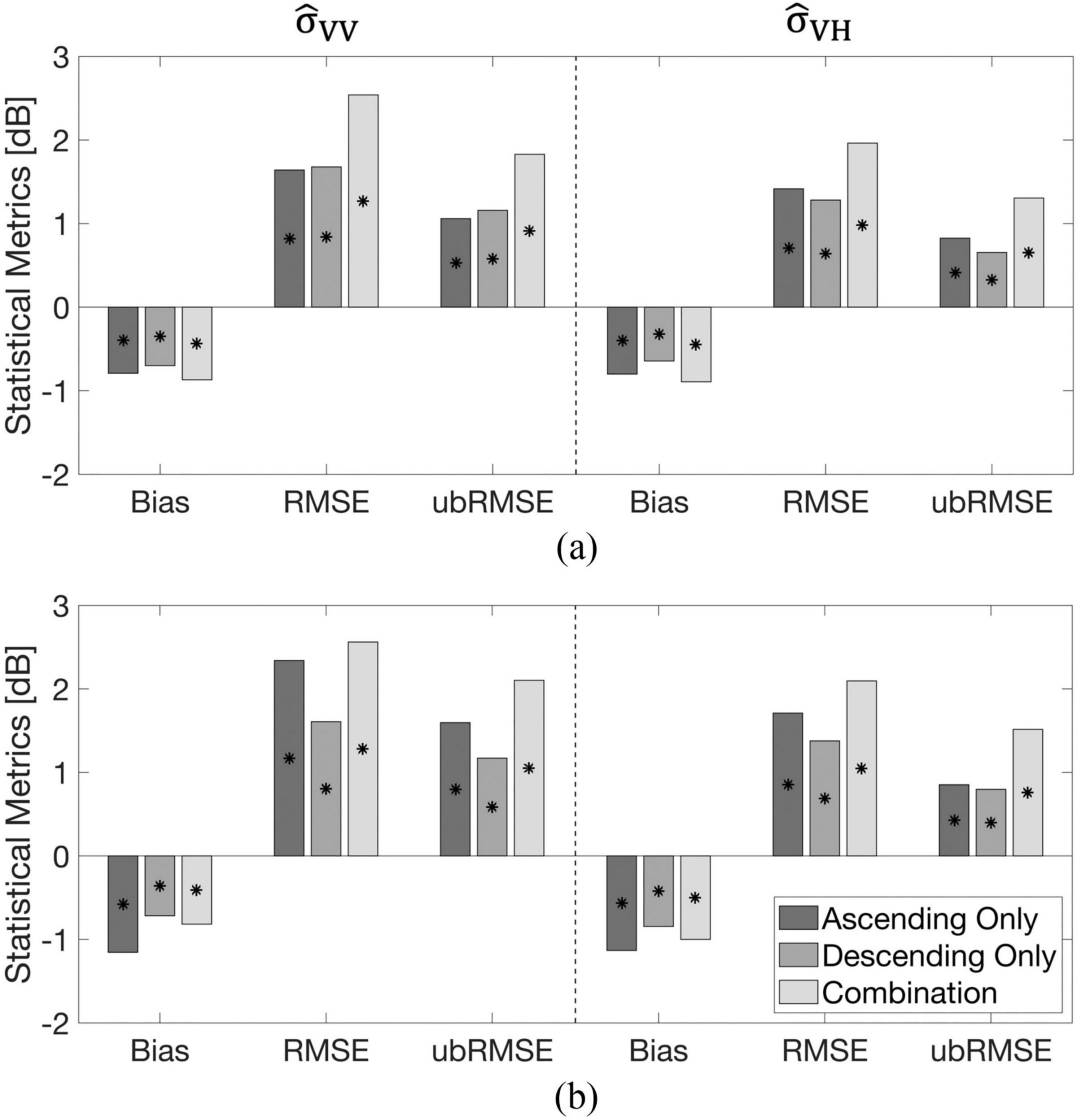
Domain-averaged statistics of predicted backscatter for three different training target sets during the (a) snow accumulation (December, January, and February) period and (b) snow ablation (March, April, and May) period. Asterisks indicate statistically significant differences between all pairs using the Wilcoxon signed rank sum test (p *<* 0.05).

Fig. 6 summarizes the domain-averaged statistics during the snow accumulation and snow ablation periods. By comparing the two different periods, the accuracy of predicted backscatter during the snow ablation (wet) period showed more negative bias than the snow accumulation (dry) period. The magnitude of bias during the snow accumulation period ranged from −0.93 to −0.64 dB and was less negative than for the snow ablation period that ranged from −1.15 to −0.72 dB. Different statistical results during the snow accumulation and snow ablation periods are associated with different physical characteristics of the snow at different times of the year. Namely, the snowpack during the ablation period tends to have deeper snow with more complex snow stratigraphy, more depth hoar, and more internal ice layers. The presence of more internal ice layers introduces even more heterogeneity, which makes the electromagnetic response of the snowpack even more complex and diverse.

In terms of different training target sets, the descending-only set yielded a slightly smaller absolute bias, RMSE, and ubRMSE at both polarizations than did the ascending-only set or the combined set during both the snow accumulation and snow ablation periods (Fig. 6). Statistics from the descending-only training set yielded a modest bias (ranged from −0.84 to −0.64 dB), RMSE (ranged from 1.28 to 1.68 dB), and ubRMSE (ranged from 0.65 to 1.17 dB) relative to the other training target sets. Different statistical results between the ascending- and descending-only overpasses resulted from the different observation times. As earlier mentioned in Section III-A, different observation times for ascending and descending overpasses resulted in different electromagnetic responses associated with the diurnal melting and refreezing cycle. More specifically, wet snow is more likely to be found on the snow surface associated with relatively warmer temperature and incoming solar radiation. This can result in large variations in surface roughness and dielectric constant, and in turn, lead to increased uncertainty of predicted backscatter using backscatter observations from ascending-only overpasses. Comparing the statistics during the snow accumulation and ablation period, predicted backscatter during the snow ablation period revealed more uncertainties than during the snow accumulation period.

In the case of the combined training set, ubRMSE was relatively large during both the snow accumulation (1.84 dB for ${\hat{\sigma }}_{VV}^{0}$ and 1.31 dB for ${\hat{\sigma }}_{VH}^{0}$) and the snow ablation (2.10 dB for ${\hat{\sigma }}_{VV}^{0}$ and 1.56 dB for ${\hat{\sigma }}_{VH}^{0}$) periods (Fig. 6). Similar findings were found in previous studies (e.g., Varade *et al.* [35], Nagler and Rott [97]) that the use of backscatter observations from both ascending and descending overpasses resulted in increased uncertainty. This phenomenon was likely due to the combination of observations from ascending and descending overpasses, which reflects differences in daytime versus nighttime snowpack conditions, and, in turn, introduces more random errors in conjunction with differences in viewing geometry between the two overpasses.

### B. Influence of Training Window Length on SVM Prediction

**Fig. 7.**
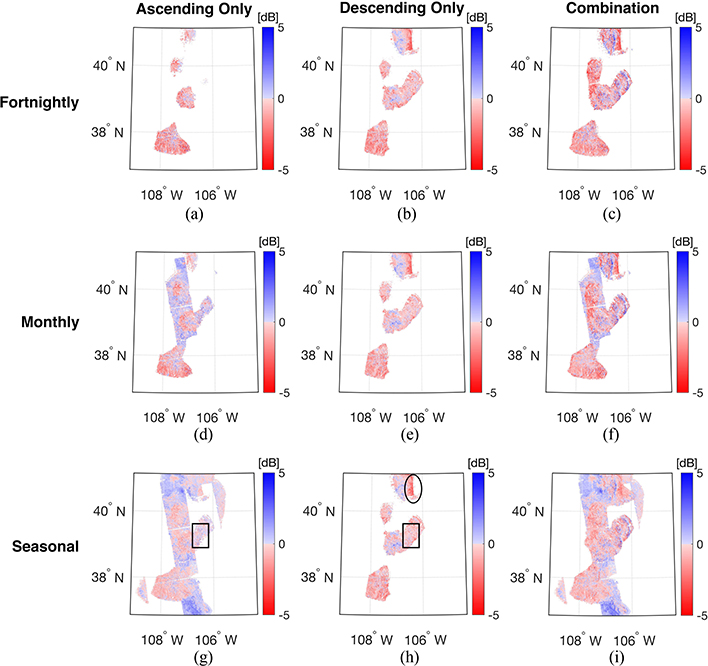
Spatial distribution of bias for co-polarized backscatter $\left({\hat{\sigma }}_{VV}^{0}\right)$ during the validation period from Sep. 2016 to Aug. 2017. The different columns represent the different training target sets: ascending-only (left column), descending-only (middle column), and combination of ascending and descending (right column). The different rows represent the different training windows (fortnightly, monthly, and seasonal from the top to bottom).

Fig. 7 illustrates the spatial distribution of bias calculated by comparing the predicted backscatter, ${\hat{\sigma }}_{VV}^{0}$, against observations *not* used during training for the three different windows *and* three different sets of training targets across the validation period. For the different sets of training targets, the spatial coverage tends to increase in accordance with the elongation of the training window. That is, when more training data is available as a function of time (and space by association), more SVMs are available to make predictions across the study domain, and hence, the increased spatial coverage. Among the three different sets of training targets, the ascending-only and the combined sets showed a significant increase in spatial coverage relative to the descending-only set. For example, spatial coverage was increased from 7.18% (fortnightly) to 30.1% (seasonal) for the ascending-only sets. Similar behavior was observed for the combined sets in that fortnightly and seasonal training showed the lowest (15.3%) and highest (33.9%) spatial coverage, respectively. As the training window is elongated, it is possible to generate SVMs at more locations due to the increased availability of training data, which resulted in expanding the spatial coverage of predicted backscatter. However, SVM prediction with the descending-only training set did not show a significant increase in spatial coverage with respect to the elongation of the training window. The fortnightly, monthly, and seasonal training period yielded a spatial coverage of 11.9%, 12.2%, and 12.2%, respectively. This behavior was related to the limited number of descending observations from Sentinel-1. Within our study area, the descending overpasses have significant data gaps prior to May 2017 based on the evolving Sentinel-1 (operational) observation scenario dictated by ESA. More specifically, most of the available dual-polarized observations in IW mode were only available for the ascending overpass before May 2017, which limits the training period to only the 2017–2018 winter season for generating SVMs using the descending-only training set. This operational issue prior to may 2017 is the cause of the limited increase in amount of spatial coverage for the descending-only training activities. Differences in the number of available training datasets also resulted in differences in the domain-averaged bias. Focusing on Fig. 7(g) and (h), the average bias within the region outlined by the black box was computed as −0.18 and −0.30 dB for the ascending-only and descending-only overpasses, respectively. At these locations, more than 30 observations were available for the ascending-only overpasses while only a maximum of 12 observations were available for the descending-only overpasses. Similarly, the area outlined by the black ellipse in Fig. 7(h) yielded a large magnitude of negative bias (approximately −5 dB) when only 10 (or fewer) descending observations were available for use during training activities. In general, as more observations were made available for use during the training procedure, a less biased result was obtained regardless of the amount of random error contained therein.

An elongation of the training window resulted in a reduction in the magnitude of bias. For example, elongating the training window from fortnightly to seasonal resulted in reducing the magnitude of bias in the southwestern and middle portions of the study area (Fig. 7). However, comparing Fig. 7(a) with Fig. 7(g) and Fig. 7(c) with Fig. 7(i), the bias in the northwestern and southern regions of the study domain using seasonal training showed a relatively larger, positive bias (approximately 4–5 dB) than in other parts of study area. Notably, the increase of the training window expands the size of the training matrix, which enables the existence of more SVMs across the study domain. However, it can also result in introducing more sources of uncertainties along with a more diverse electromagnetic response of the snowpack. For example, Sentinel-1 observations during the spring (March, April, and May) and Fall (September, October, and November) were utilized to predict backscatter during the winter season. During the Fall season, the snowpack is relatively shallow and relatively dry whereas the snowpack during the Spring season is typically deeper, includes more depth hoar, and contains more internal ice layers along with wind slab. Accordingly, this mixed information content of dry, shallow snow with deeper, wetter snow in a single training matrix provides a complicated, mixed electromagnetic response that can be difficult for the SVM to reproduce, and hence, results in degrading the prediction accuracy. This further motivates the physically constrained ML approach of delineating dry versus wet snow conditions, for example, that is more implicit in the fortnightly and monthly training approaches.

**Fig. 8.**
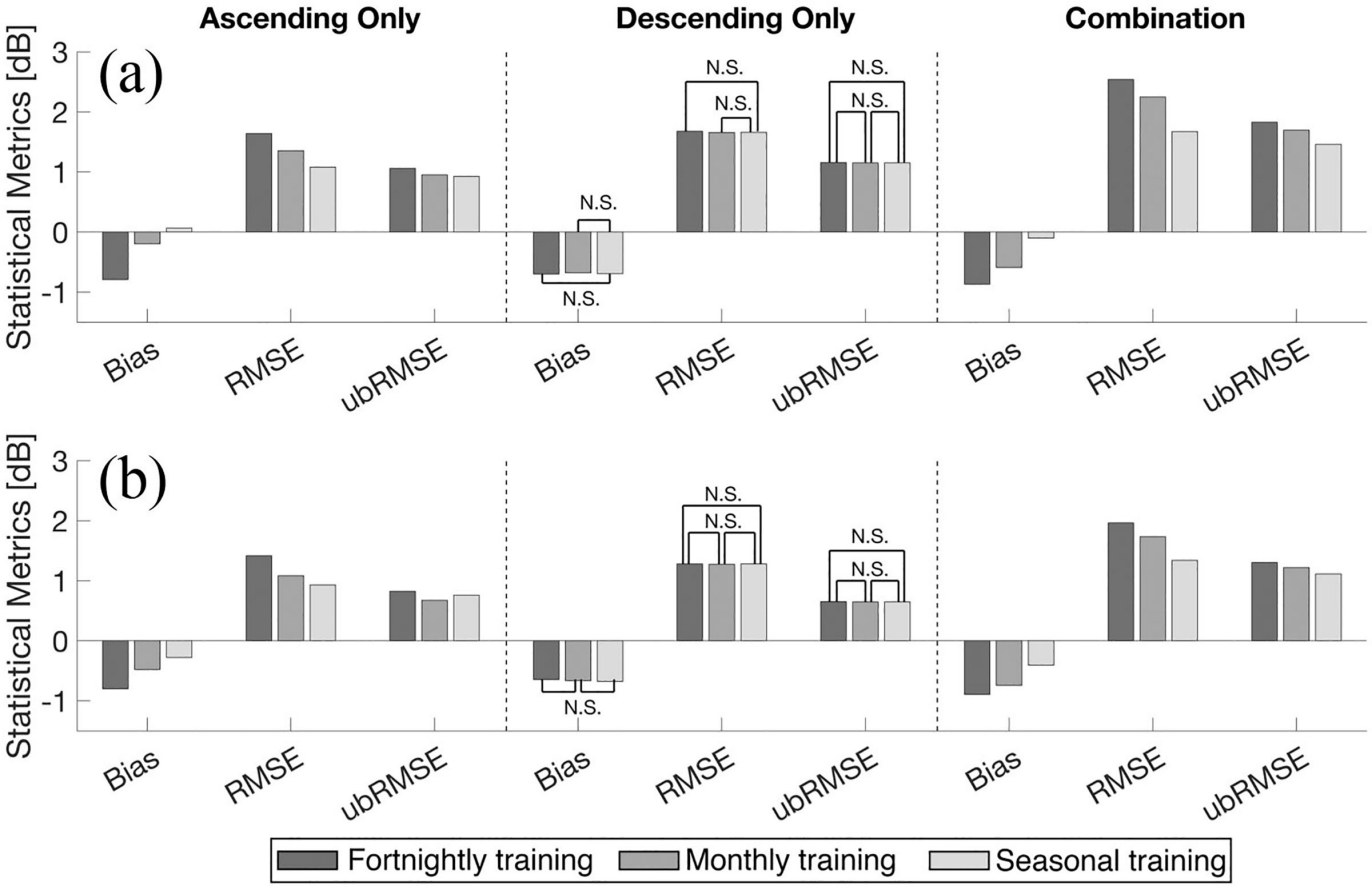
Summary of domain-averaged statistics (e.g., bias, RMSE, and ubRMSE) of predicted backscatter for fortnightly, monthly, and seasonal training windows during the snow accumulation (December, January, and February) period. N.S. represents no statistically significant difference between pairs at *p* = 0.05 using the Wilcoxon signed rank sum test. Other datasets achieved statistically significant differences at *p* = 0.05 if not marked. Left-most column used ascending-only training targets; middle column used descending-only training targets; right-most column used a combination of the two. The different bar colors represent different training window lengths.

**Fig. 9.**
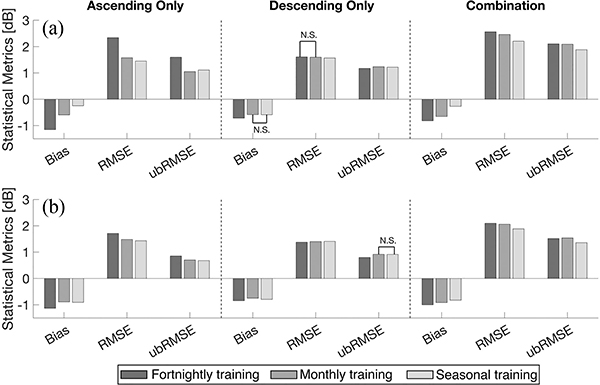
Same as Fig. 8 except for the snow ablation (March, April, and May) period.

Figs. 8 and 9 describe the domain-averaged statistics of predicted backscatter at both co- and cross-polarization using the three different training periods and three different sets training targets during the snow accumulation and ablation periods, respectively. During the snow accumulation period, the magnitude of all the statistics (e.g., bias, RMSE, and ubRMSE) showed improvement, in general, with a lengthening of the training window (Fig. 8). Bias for predicted co-polarized backscatter, ${\hat{\sigma }}_{VV}^{0}$, became less negative from −0.87 dB for fortnightly training, −0.59 dB for monthly training, and −0.10 dB for seasonal training. Even though the types of snow (e.g., shallow versus deep, dry versus wet) are more complex as more data is included over a longer time window, these results illustrate the impact of how *more* training data often yields a more robust SVM with a temporally sparse training set.

Statistics during the snow ablation period showed a similar behavior during the snow accumulation period in that most of the domain-averaged statistics showed a decreasing trend among the three different sets of training targets as the training window was elongated (Fig. 9). At the same time, however, the snow ablation period sometimes yielded an opposite behavior indicating that statistics were worse when elongating the training window length. For instance, the magnitude of bias from predicted ${\hat{\sigma }}_{VH}$ using the ascending- and descending-only training sets was decreased from fortnightly (−1.13 dB for ascending-only and −0.84 dB for descending-only training set) to monthly training (−0.89 dB for ascending-only and −0.75 dB for descending-only training set) while it was increased for seasonal training (−0.91 dB for ascending-only and −0.80 dB for descending-only training set) even though seasonal training provides the largest set of training data at a given location. Further, ubRMSE for the ascending-only training set for ${\hat{\sigma }}_{VV}^{0}$ and descending-only training set for ${\hat{\sigma }}_{VV}^{0}$ and ${\hat{\sigma }}_{VH}^{0}$ showed a slight increasing trend as the training window was elongated (Fig. 9). These phenomena suggest that a mixture of signals during different snow conditions within a single training matrix resulted in introducing more random errors to the predicted backscatter. This infers a conundrum of more training data versus “better” training data when training targets are sparse in space and time. In the limit, as the number of training data approaches infinity, the physically constrained approaches should be superior, but is not always evident given the severity of the data sparsity in this study.

### C. Influence of Separate Training for Dry and Wet Snow Conditions

C-band backscatter has distinct characteristics related to dry versus wet snow conditions as mentioned in Section III-A. Statistical analysis of predicted backscatter using different sets of training targets and training window lengths highlighted that including signals from both dry and wet snow conditions at the same time resulted in a degradation of SVM performance. Here, the influence of different snow liquid water content toward SVM prediction is *explicitly* examined by comparing the statistics of predicted backscatter with and without explicit dry snow and wet snow delineation based on Noah-MP estimates. Previous research utilized the diurnal amplitude variation and cross-polarization gradient ratio based on PMW observations for detecting snowmelt. In this study, however, snow liquid water content from Noah-MP output was utilized for delineating dry snow versus wet snow. Here, a pixel with more than 0% liquid water content was classified as wet snow based on the general classification in Fierz *et al.* [98]. It is noted here that Noah-MP snow liquid water content estimates are far from perfect. However, in the absence of *in situ* measurements of liquid water content, it is assumed here that the model-based estimates are a viable proxy. Similar to the previous sections, ascending-only, descending-only, and combined training sets were used separately.

**Fig. 10.**
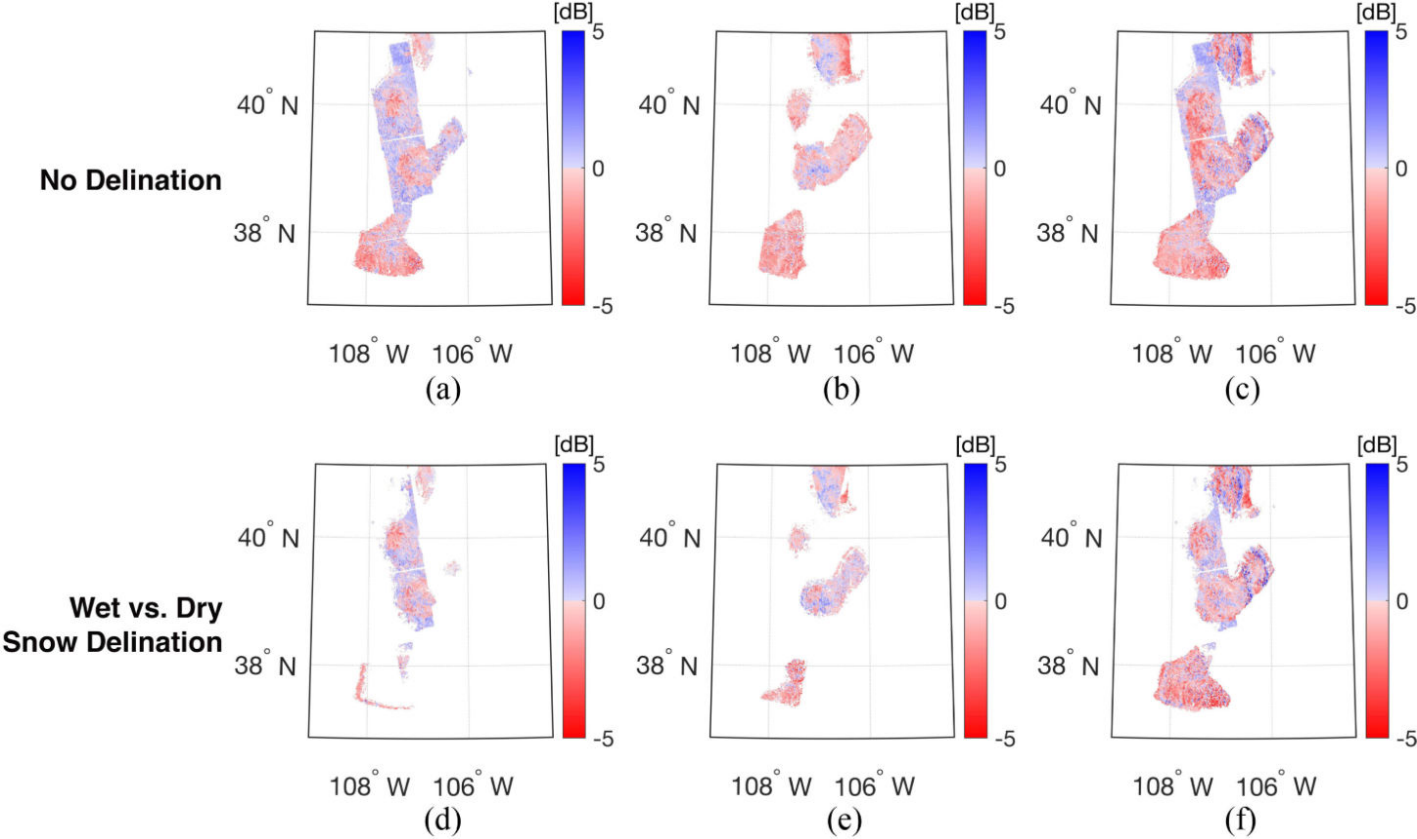
Spatial distribution for bias of predicted ${\hat{\sigma }}_{VV}^{0}$ without explicit dry versus wet snow delineation (top row) and with explicit dry versus wet snow delineation (bottom row) from Sep. 2016 to Aug. 2017. The different columns represent the training sets for ascending-only (left column), descending-only (middle column), and combination of ascending and descending (right column). The increase in white space in the bottom row (relative to the top row) is due to fewer training targets being available, and hence, fewer SVMs that can be generated due to increased data sparsity.

The spatial distributions of bias of the predicted co-polarized backscatter, ${\hat{\sigma }}_{VV}^{0}$, using the three different training sets without and with dry snow versus wet snow delineation via Noah-MP are shown in Fig. 10. Wet snow is defined here as simply as when the snow liquid water content was greater than zero. Comparing Fig. 10(a)–(c) with Fig. 10(d)–(f), bias was modestly reduced when the separate SVMs for dry and wet snow pixels were generated. The use of modeled liquid water content from Noah-MP added another physical constraint during SVM training. As such, the size of the training matrix was further reduced, which resulted in fewer trained SVMs that in turn reduced the spatial coverage. However, Table IV showed that separate training for dry and wet snow resulted in improving most of the domain-averaged statistics of predicted backscatter at both polarizations in spite of the reduced number of targets for use during training. For the ascending-only and descending-only training sets, the bias, RMSE, and ubRMSE were slightly improved only when using the explicit dry versus wet snow delineation during training. Even though the combination training set also showed slight improvement in ubRMSE, the RMSE was slightly increased when using the explicit dry versus wet delineation during training. This phenomenon was due, in large part, to the different observation times for ascending and descending overpasses. As mentioned earlier, the different observations at different times (overpasses) will often have different snow conditions depending on the diurnal melting and refreezing cycle. Accordingly, even if the specific pixel is classified as wet or dry snow pixel based on the modeled snow liquid water content, the combined overpass training set is often composed of a mixture of both wet snow and dry snow signals.

**Fig. 11.**
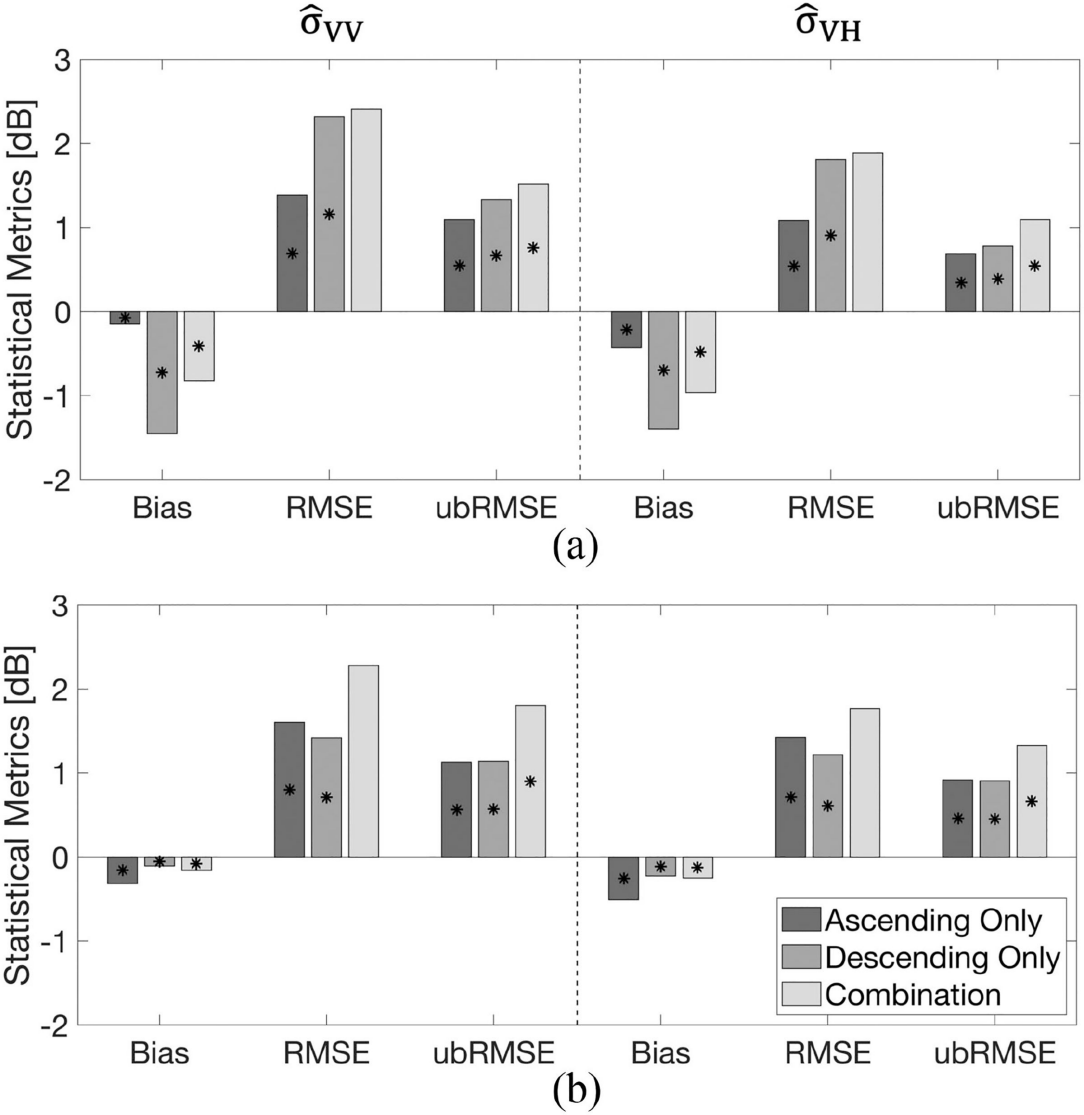
Domain-averaged statistics of predicted backscatter for three different training approaches at (a) dry snow locations and (b) wet snow locations during the validation period of Sep. 2016 to Aug. 2017. Asterisks indicate statistically significant differences between all pairs using the Wilcoxon signed rank sum test (*p <* 0.05).

Statistics of predicted backscatter based on *explicit* dry versus wet snow delineation were classified into dry and wet snow pixels based on the amount of liquid water content and analyzed in order to evaluate the efficacy of the physically constrained SVM (Fig. 11). The results showed that predicted backscatter over dry snow and wet snow pixels using ascending-only training exhibited comparable statistical performance. For example, ${\hat{\sigma }}_{VV}^{0}$ over dry snow pixels yielded RMSE and ubRMSE of 1.38 dB and 1.09 dB, respectively, which were similar for wet snow pixels (1.37 dB for RMSE and 1.13 dB for ubRMSE). Similarly, bias and RMSE of ${\hat{\sigma }}_{VH}^{0}$ over dry snow pixels was −0.43 and 1.08 dB, respectively, which showed similar statistical performance as the wet snow pixels (−0.51 dB for bias 1.09 dB for RMSE). As mentioned earlier, ascending overpasses have relatively wetter surface snow conditions due to the small amount of diurnal melting during the afternoon. It is believed that this leads to the similar statistical behavior over dry snow pixels versus wet snow pixels using the ascending-only training set.

In the case of descending-only and combined training sets, wet snow pixels showed a lower magnitude of bias than did dry snow pixels. Bias of ${\hat{\sigma }}_{VV}^{0}$ and ${\hat{\sigma }}_{VH}^{0}$ using the descending-only training set was −1.45 and −1.40 dB for dry snow while it was reduced to −0.11 and −0.23 dB during wet snow conditions. In general, C-band backscatter has a greater penetration depth for dry snow (~20 m) than wet snow (~3 cm) [49], [68], [99]. Furthermore, C-band backscatter during dry snow conditions, in general, is dominated by backscatter at the snow–land interface as other components (i.e., volume scattering or air–snow interface scattering) are relatively small [70]. This behavior resulted in no significant difference between backscatter from snow-free conditions versus shallow, dry snow conditions [45], but that volume scattering (and hence backscatter) can still be significantly modulated during deep, dry snow conditions [46]. Conversely, backscatter during wet snow conditions is generally dominated by either scattering at the air–snow surface or by volume scattering depending on the snow liquid water content [70]. Hence, the backscatter has a relatively larger variability during wet snow conditions as C-band radiation undergoes a large amount of absorption and/or reflection (Fig. 2). This increased sensitivity during wet snow conditions provides more information content for the SVM to yield better predictions regarding C-band backscatter (and its relation to snow mass) as compared to the SVM predictions during dry snow conditions when C-band backscatter is predicated more on backscatter from the snow–land interface rather than volume scattering associated with terrestrial snow mass. It is hypothesized that these differences in the fundamental physics result in better statistical performance as in this case related to snow mass when the snow is wet rather than dry.

## V. Conclusion

The main goal of this article is to assess the feasibility of physically constrained SVMs to predict C-band backscatter over snow-covered terrain in Western Colorado. More specifically, the influence of different training target sets, training window lengths, and dry versus wet snow delineation on SVM efficacy were considered in conjunction with the first-order electromagnetic response of snow.

Results indicated that a combination of backscatter observations from ascending and descending overpasses allows for the usage of more available training data (in time and space) during SVM training activities. This results in a significant increase in spatial extent and decrease of the domain-averaged bias of predicted backscatter. However, this approach degraded the RMSE and ubRMSE due to the mixture of different signals associated with different times of day and different viewing geometries during the different overpasses. Similarly, elongation of the training window length also yielded an increase in the spatial coverage of predicted backscatter and a decrease in the domain-averaged bias, but introduced more random errors due to the mixture of signals from different snow conditions.

Finally, separate training of dry and wet snow pixels using modeled snow liquid water content from Noah-MP yielded a statistical improvement in reducing the magnitude of bias, RMSE, and unbiased RMSE of ascending-only and descending-only training sets. Moreover, separate training for dry versus wet snow pixels, and the physical constraints associated with the different electromagnetic responses of the snow, demonstrated more robustness at wet snow locations than dry snow locations. This implies that C-band backscatter showed relatively higher sensitivity toward wet snow than dry snow due to the different electromagnetic responses (e.g., scattering versus absorption).

In summary, prediction of C-band backscatter over snow-covered land using a physically constrained ML approach suggests the necessity of considering the first-order physics during ML training in order to ensure the ML algorithm produces the right answer for the right reason. As part of a future follow-on study, it is recommended that the inclusion of local incidence angle obtained through preprocessing with Sentinel Application Platform (SNAP) provided by ESA for use as an input to SVM regression should be explored in detail, including the impact on SVM regression prediction performance over the snow-covered terrain. Further, this article provides a fundamental framework utilizing SVM regression as an observation operator within a data assimilation system to be pursued in a follow-on study in order to improve model-derived snow mass information based on a Bayesian merger of an advanced land surface model with C-band backscatter observations.

## Supplementary Material

supp1-3053945

## Figures and Tables

**TABLE I:** Main Characteristics of Sentinel-1 IW Ground-Range Detected (GRD) Products

**Product Type**	GRD-High
**Center Frequency**	5.407 GHz
**Swath Width**	250 km
**Incidence Angle**	29.1° – 46.0°
**Resolution**	20 m × 22 m
**Pixel Spacing**	10 m × 10 m

**TABLE II:** SVM Inputs and Outputs

**Inputs**	**Unit**	**Scale Factor**
Snow Water Equivalent	m	10
Snow Density^a^	kg m^−3^	0.01
Snow Liquid Water Content^a^	mm	1
Top layer Snow Temperature	K	0.01
**Outputs**	**Unit**	**Scale Factor**
${\hat{\sigma }}_{VV}^{0}$	dB	none
${\hat{\sigma }}_{VH}^{0}$	dB	none

a Column-integrated estimates.

**TABLE III:** Domain-Averaged Statistics of Predicted Backscatter From Ascending-Only, Descending-Only, and the Combination of Both Ascending and Descending Overpasses Compared Against the Sentinel-1 Observations From Sep. 2016 to Aug. 2017 *Not* Used During Fortnightly Training

Datasets	**Bias**		**RMSE**		**ubRMSE**		**Spatial Coverage**
	${\hat{\sigma }}_{VV}^{0}$	${\hat{\sigma }}_{VH}^{0}$	${\hat{\sigma }}_{VV}^{0}$	${\hat{\sigma }}_{VH}^{0}$	${\hat{\sigma }}_{VV}^{0}$	${\hat{\sigma }}_{VH}^{0}$	[%]

**Ascending-only**	−0.89	−0.84	1.64	1.36	0.91	0.73	7.2
**Descending-only**	−0.74	−0.82	1.58	1.36	1.16	0.80	11.9
**Combination**	−0.83	−0.95	2.54	2.06	2.08	1.54	15.2

*Note:* All statistics are different at p = 0:05 using the Wilcoxon signed rank sum test.

**TABLE IV:** Domain-Averaged Statistics of Predicted Backscatter Using the Three Different Training Sets (a) Without Dry Versus Wet Snow Classification and (b) With Dry Versus Wet Snow Classification Based on Noah-mp

(a)	**Bias [dB]**		**RMSE [dB]**		**ubRMSE [dB]**	
Datasets	${\hat{\sigma }}_{VV}^{0}$	${\hat{\sigma }}_{VH}^{0}$	${\hat{\sigma }}_{VV}^{0}$	${\hat{\sigma }}_{VH}^{0}$	${\hat{\sigma }}_{VV}^{0}$	${\hat{\sigma }}_{VH}^{0}$

**Ascending-only**	−0.16	−0.48	1.38	1.12	1.03	0.73
**Descending-only**	−0.60	−0.72	1.58	1.36	1.23	0.90
**Combination**	−0.48	−0.72	2.22	1.77	1.86	1.36

(b)	**Bias [dB]**		**RMSE [dB]**		**ubRMSE [dB]**	
Datasets	${\hat{\sigma }}_{VV}$	${\hat{\sigma }}_{VH}$	${\hat{\sigma }}_{VV}$	${\hat{\sigma }}_{VH}$	${\hat{\sigma }}_{VV}$	${\hat{\sigma }}_{VH}$

**Ascending-only**	−0.12	−0.39	1.37	1.09	1.05	0.69
**Descending-only**	−0.30	−0.40	1.52	1.23	1.16	0.88
**Combination**	−0.54	−0.67	2.30	1.78	1.59	1.14

*Note:* All statistics are different at p = 0:05 using the Wilcoxon signed rank sum test.

## Footnotes

1 [Online]. Available: https://data.fs.usda.gov/geodata/rastergateway/forest_type

## Appendix A

### Support Vector Regression

Assume a [*M* × *N*] training matrix, *x*, such that it contains *N =* 4 different geophysical variables simulated from Noah-MP for characterizing the physical conditions of snow at *M* different times for a given location in space. Training target (*z*; Sentinel-1 backscatter observations in this article) has a size of [*M* × 1]. The objective function for SVM regression can be written as

(5)
f(ω,δ)=〈w⋅ϕ(x)〉+δ

where *ω* is a weighting factor and *ϕ*(*x*) is a nonlinear function for mapping the geophysical variables into observation (i.e., backscatter) space. ⟨*ω · ϕ*(*x*)⟩ refers the inner dot product of *ω* and *ϕ*(*x)*. As the main goal of SVM regression is to optimize parameters to increase the accuracy of *f*(*ω, δ)*, the basic formulation of nonlinear SVM regression can be expressed as follows:

(6)
$$\mathrm{minimize}\;\frac{1}{2}\|w{\|}^{2}+C\sum_{i=1}^{m}\left(\xi_{i}+\xi_{i}^{*}\right)\;\;\text{subject}\;\text{to}\;\{\begin{array}{l} f(\omega ,\delta )-z_{i}\leq \varepsilon +\xi_{i}\\ z_{i}-f(\omega ,\delta )\leq \varepsilon +\xi_{i}^{*}\\ \xi_{i},\xi_{i}^{*}\geq 0 \end{array}$$

where *C(*> 0) is the user-defined constant representing the trade-off between tolerance of *ε* and *f*(*ω, δ*) [100]. *ξ*_*i*_ and $\xi_{i}^{*}$ are slack variables and *z*_*i*_ represents the Sentinel-1 backscatter observation at timestep *i*. Optimization of (6) is commonly regarded as a dual optimization problem [89] and can be solved by applying a dual set of Lagrangian multipliers (*α*_*i*_ and $\alpha_{i}^{*}$) as

(7)
$$\begin{array}{l} \mathrm{minimize}\;\frac{1}{2}\sum_{i,j=1}^{m}\left(\alpha_{i}-\alpha_{i}^{*}\right)\left(\alpha_{j}-\alpha_{j}^{*}\right)\langle \phi \left(x_{i}\right)\cdot \phi \left(x_{j}\right)\rangle \\ \;+\sum_{i=1}^{m}\varepsilon \left(\alpha_{i}+\alpha_{i}^{*}\right)-\sum_{i=1}^{m}z_{i}\left(\alpha_{i}+\alpha_{i}^{*}\right)\\ \;\text{subject}\;\text{to}\;\{\begin{array}{l} \sum_{i=1}^{m}\varepsilon \left(\alpha_{i}+\alpha_{i}^{*}\right)=0\\ \alpha_{i},\alpha_{i}^{*}\in [0,C],i=1,2,\ldots ,m \end{array}. \end{array}$$

When the dual Lagrange multipliers yield a nonzero value, then it is the so-called support vector that lies on the margins [100].

In real-world applications, computation of ⟨*ϕ*(*x*_*i*_) *· ϕ*(*x*_*j*_)⟩ can be too computationally costly, which motivates one to employ a kernel technique for improving computational efficiency by directly mapping the solution into higher dimensional space [92], [100]. There are different types of kernel functions that can be used such as linear, nonlinear, and polynomial forms [101]. Among them, the radial RBF is employed in this study due to its advantages in dealing with datasets having nonlinear relationships between inputs and outputs (training target). As such, the kernel function can be expressed as

(8)
$$k\left(x_{i},x_{j}\right)=\langle \phi \left(x_{i}\right),\phi \left(x_{j}\right)\rangle =\exp\left\{-\gamma {\left\|x_{i}-x_{j}\right\|}^{2}\right\}$$

where *x*_*i*_ and *x*_*j*_ represent a single instance of x in time and space and ∥.∥ represents the Euclidean norm between *ϕ*(*x*_*i*_) and *ϕ*(*x*_*j*_). The positive parameter, *γ*, is an adjustable parameter to control the width of the Gaussian variable. When *γ* is small, more weight will be given to the points closer to *x*_*i*_ while a larger *γ* indicates more importance to points far from *x*_*i*_.
