# Supplementary material for: Prediction of Active Microwave Backscatter Over Snow-Covered Terrain Across Western Colorado Using a Land Surface Model and Support Vector Machine Regression
Source: IEEE J Sel Top Appl Earth Obs Remote Sens. Author manuscript; Available in PMC 2022 Feb 11. (PMC8833106; doi:10.1109/jstars.2021.3053945)
Supplement: supp1-3053945 [file NIHMS1675681-supplement-supp1-3053945.docx]

Supplementary Figure for Prediction of active microwave backscatter over snow-covered terrain across Western Colorado using a land surface model and support vector machine regression


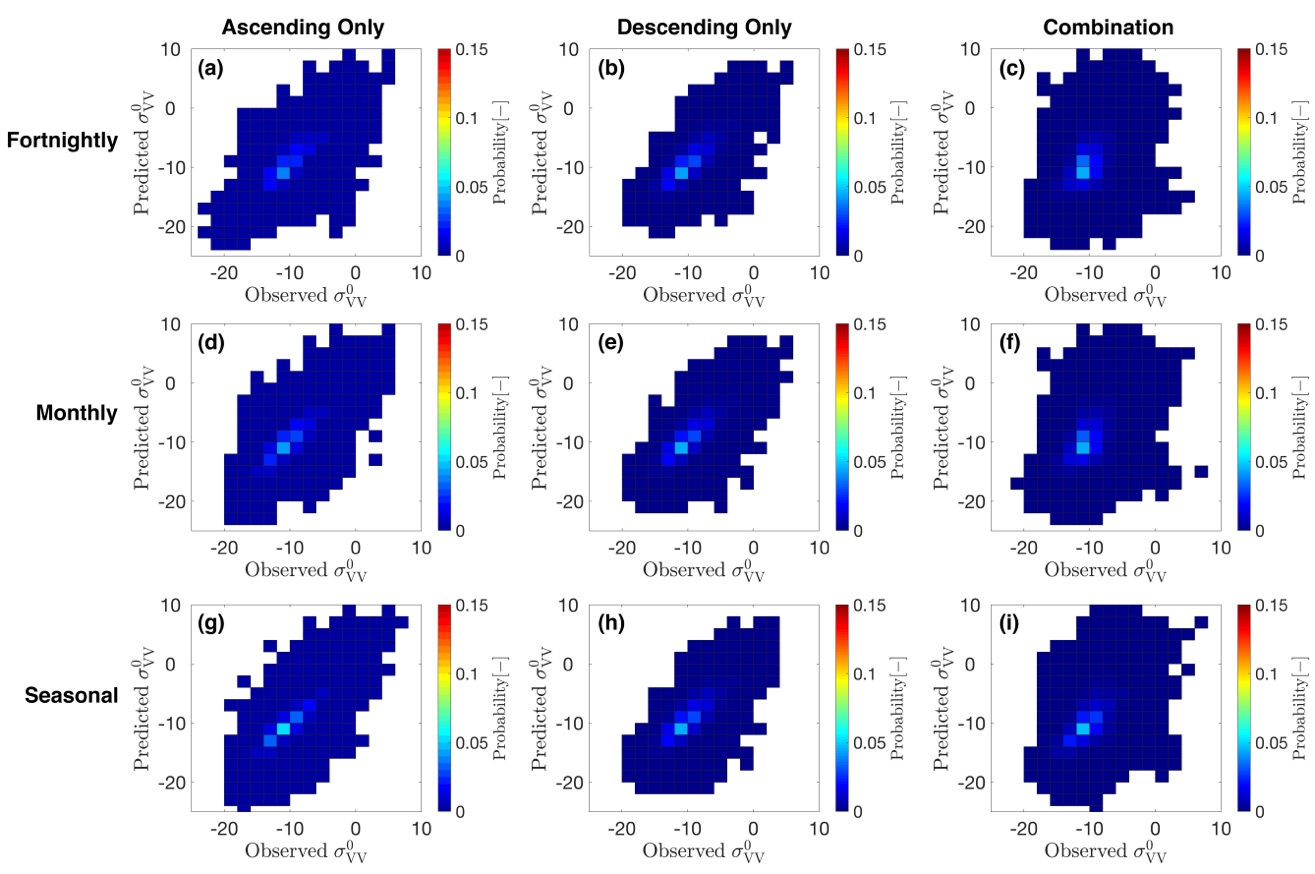


Fig. S1. Scatter plot of observed and predicted co-polarized backscatter during the validation period from Sep. 2016 to Aug. 2017. The different columns represents the different training targets: ascending-only (left column), descending-only (middle-column), and combination of ascending and descending (right column). The different rows represent the different training windows (fortnightly, monthly, and seasonal training period from the top to bottom).
